# Supplementary material for: High-fat diet in early life triggers both reversible and persistent epigenetic changes in the medaka fish (Oryzias latipes)
Source: BMC Genomics. 2023 Aug 21;24:472. doi: 10.1186/s12864-023-09557-1 (PMC10441761; doi:10.1186/s12864-023-09557-1)
Supplement: Supplementary file 1 — Additional file 1: Figure S1. Confirmation of hepatocyte-specific expression of GFP in tdo2:GFP transgenic medaka. [file 12864_2023_9557_MOESM1_ESM.pdf]

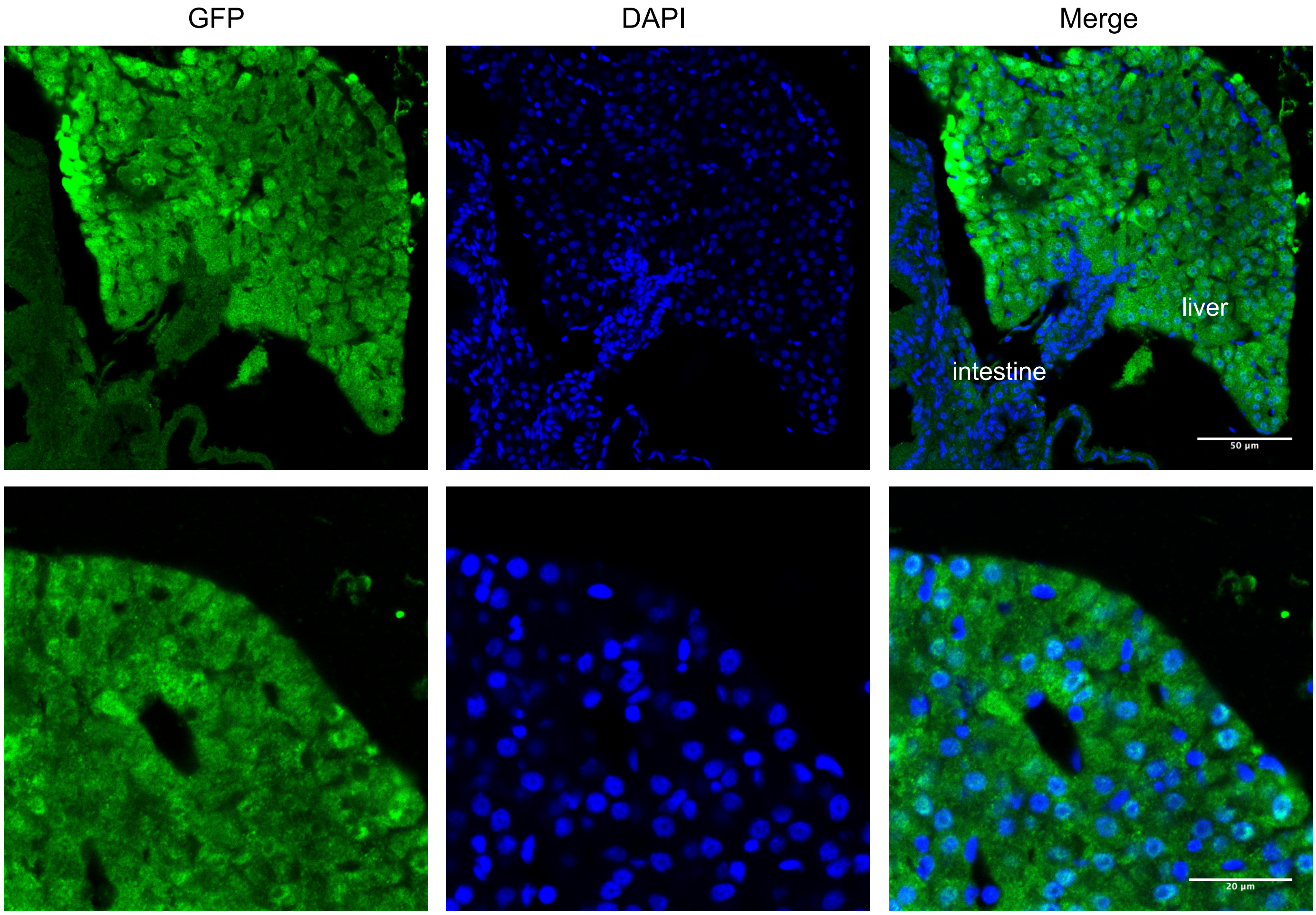

Figure S1: Confirmation of hepatocyte-specific expression of GFP in *tdo2*:GFP transgenic medaka. Immunofluorescent images of a liver section stained with GFP antibody are shown. Note that GFP signal was detected in hepatocytes, but not in red blood cells, endothelial cells in the liver, or cells in the intestine. Scale bar: 50 µm (upper), 20 µm (lower).
