# Supplementary material for: High-fat diet in early life triggers both reversible and persistent epigenetic changes in the medaka fish (Oryzias latipes)
Source: BMC Genomics. 2023 Aug 21;24:472. doi: 10.1186/s12864-023-09557-1 (PMC10441761; doi:10.1186/s12864-023-09557-1)
Supplement: Supplementary file 2 — Additional file 2: Figure S2. Sorting of GFP-positive hepatocytes from livers of tdo2:GFP medaka. [file 12864_2023_9557_MOESM2_ESM.pdf]

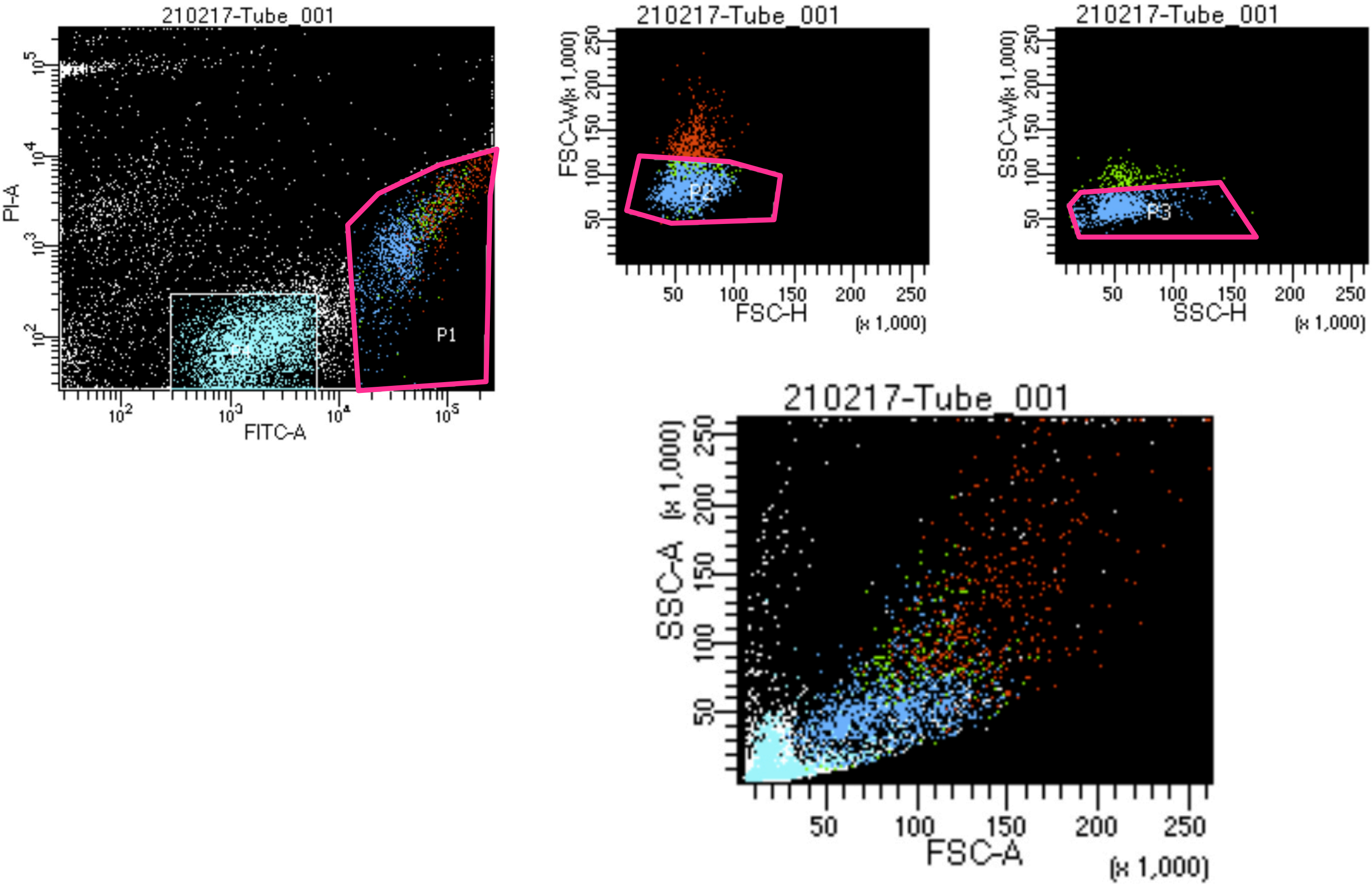

Figure S2: Sorting of GFP-positive hepatocytes from livers of *tdo2*:GFP medaka.

FACS-sorting strategy of GFP-positive hepatocytes from liver cell suspensions of *tdo2*:GFP transgenic medaka. GFP-positive, propidium-iodide (PI)-negative, singlet cells were sorted.
