# Supplementary material for: High-fat diet in early life triggers both reversible and persistent epigenetic changes in the medaka fish (Oryzias latipes)
Source: BMC Genomics. 2023 Aug 21;24:472. doi: 10.1186/s12864-023-09557-1 (PMC10441761; doi:10.1186/s12864-023-09557-1)
Supplement: Supplementary file 3 — Additional file 3: Figure S3. Differentially expressed genes/peaks at FDR < 0.1, corrected for multiple testing. [file 12864_2023_9557_MOESM3_ESM.pdf]

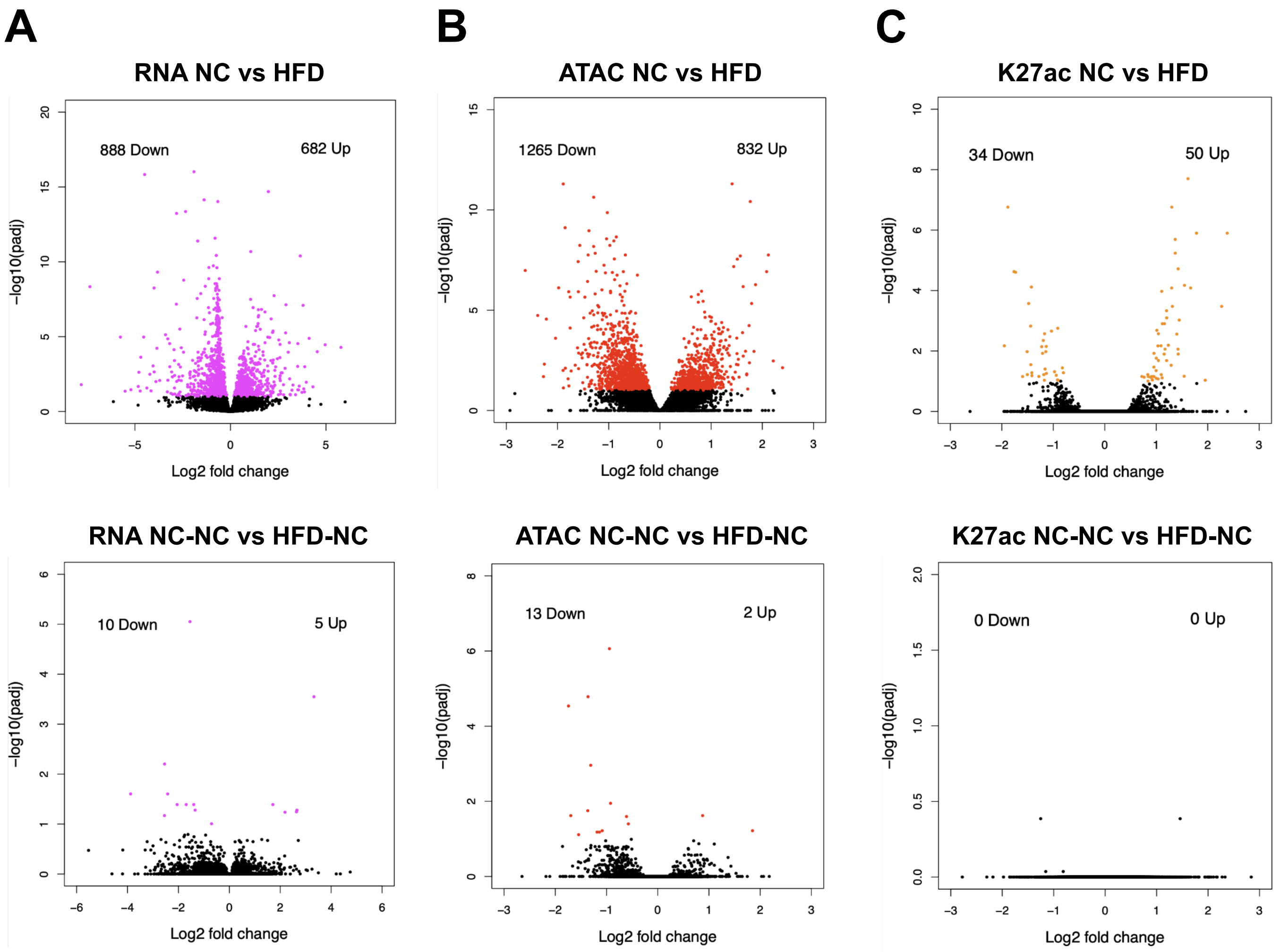

Figure S3: Differentially expressed genes/peaks at FDR < 0.1, corrected for multiple testing.

Volcano plots showing **(A)** differentially expressed genes by RNA-seq, **(B)** differentially accessible peaks by ATAC-seq, and **(C)** differentially H3K27ac-enriched peaks by ChIP-seq. X-axis: log2 fold change of normalized read counts within genes/peaks (upper, HFD/NC; lower, HFD-NC/NC-NC), y-axis: –log10(FDR).
