# Supplementary material for: High-fat diet in early life triggers both reversible and persistent epigenetic changes in the medaka fish (Oryzias latipes)
Source: BMC Genomics. 2023 Aug 21;24:472. doi: 10.1186/s12864-023-09557-1 (PMC10441761; doi:10.1186/s12864-023-09557-1)
Supplement: Supplementary file 4 — Additional file 4: Figure S4. Categorization of peaks into promoters and enhancers. [file 12864_2023_9557_MOESM4_ESM.pdf]

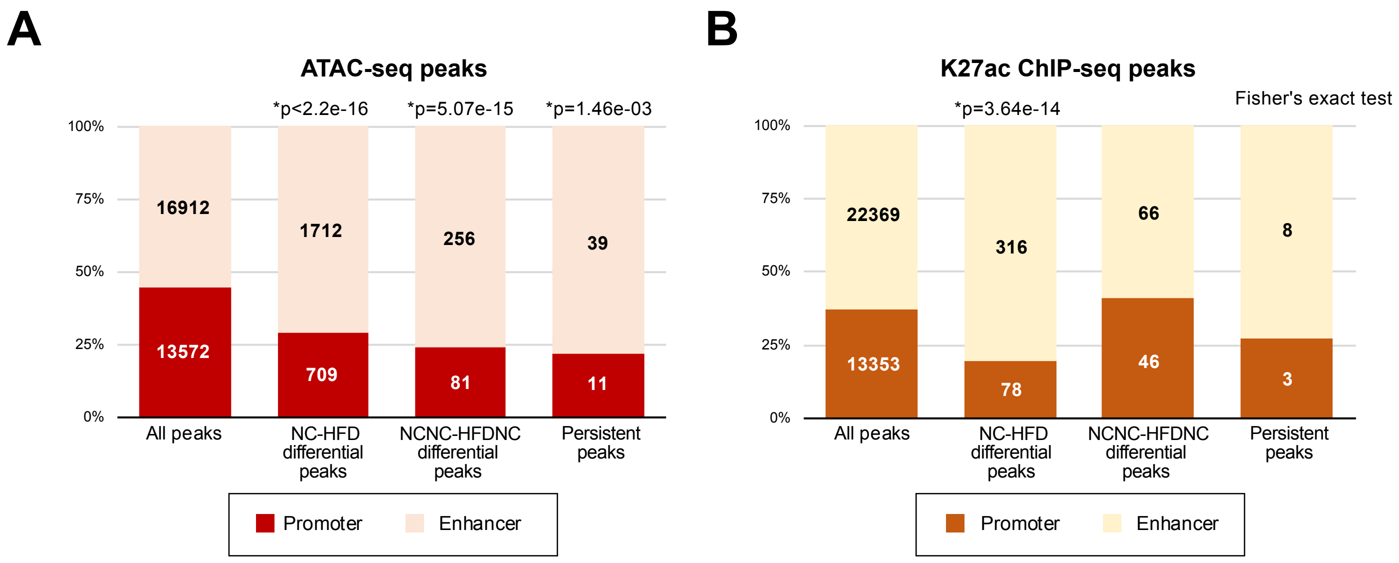

Figure S4: Categorization of peaks into promoters and enhancers.

(A) ATAC-seq peaks (30,484 peaks in total) were categorized as promoters if peaks were located inside 2 kb on either side of the transcription start sites (TSS), while others were categorized as enhancers. **(B)** H3K27ac ChIP-seq peaks (35,722 peaks in total) were categorized as promoters if peaks were located inside 2 kb on either side of the TSS, while others were categorized as enhancers. Enrichment of peaks with differential signal at enhancers was tested by Fisher's exact test.
