# Supplementary material for: High-fat diet in early life triggers both reversible and persistent epigenetic changes in the medaka fish (Oryzias latipes)
Source: BMC Genomics. 2023 Aug 21;24:472. doi: 10.1186/s12864-023-09557-1 (PMC10441761; doi:10.1186/s12864-023-09557-1)
Supplement: Supplementary file 5 — Additional file 5: Figure S5. Western blotting of H3K27ac, H3K27me3, and H3K9me3 histone modifications in livers. [file 12864_2023_9557_MOESM5_ESM.pdf]

H3K27ac

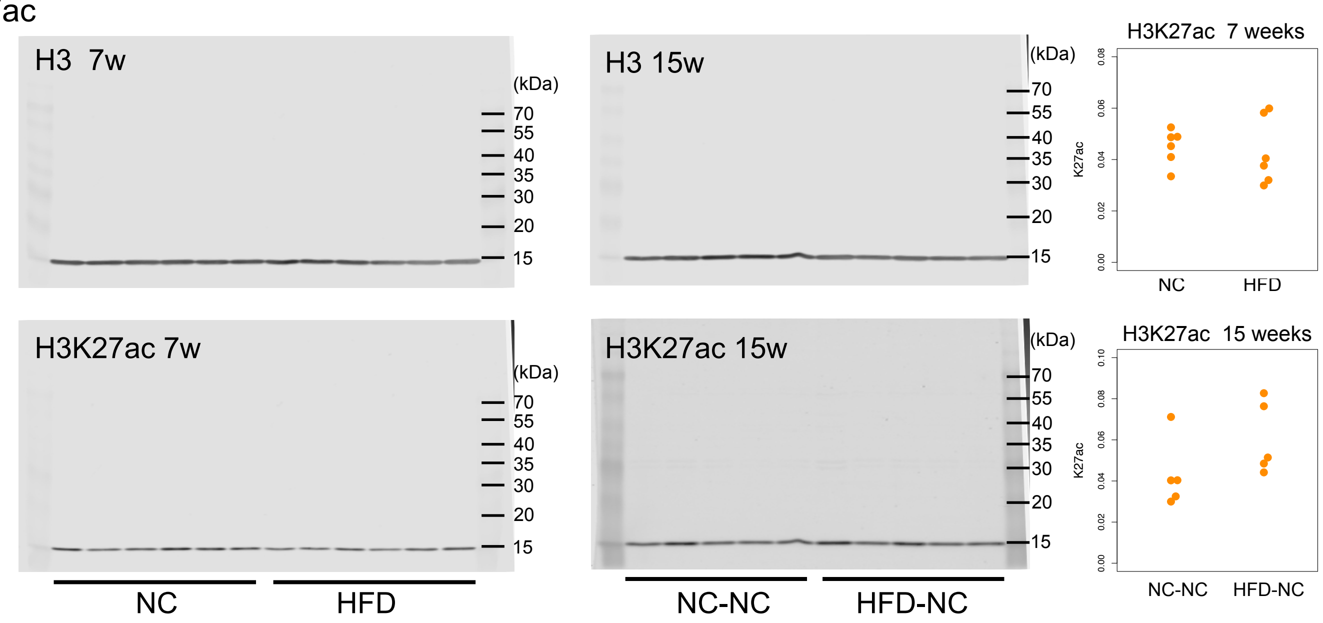

H3K27me3

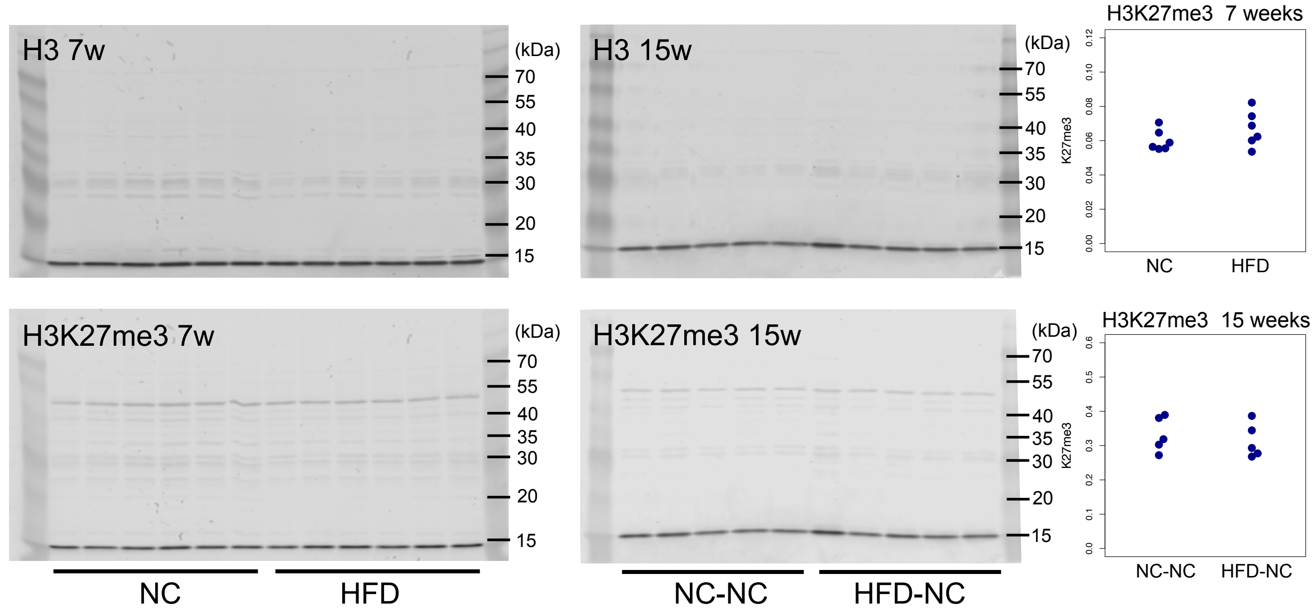

H3K9me3

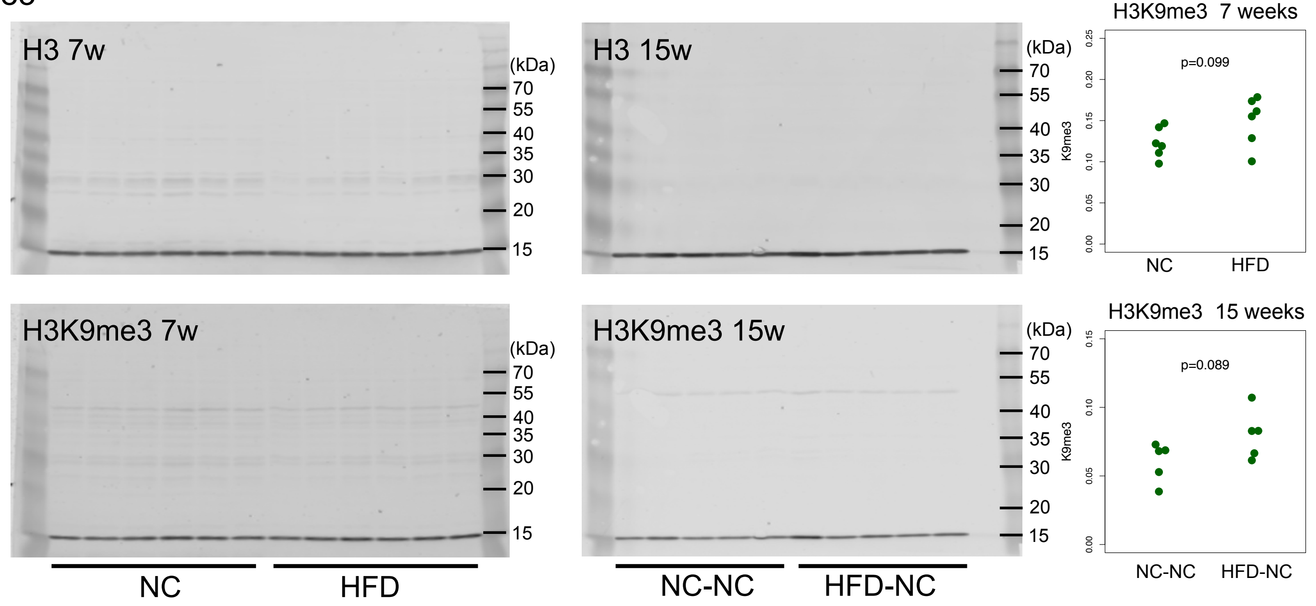

Figure S5: Western blotting of H3K27ac, H3K27me3, and H3K9me3 histone modifications in livers.

Left: Raw images of western blotting of each histone modification and histone H3. One lane indicates liver lysate of a single fish, concentration of which was adjusted by liver weight. Right: plots of signal intensities of each histone modification, normalized by signal intensities of histone H3. Note that there is little difference in the global levels of each histone modification in medaka liver between dietary conditions.
