# Supplementary material for: High-fat diet in early life triggers both reversible and persistent epigenetic changes in the medaka fish (Oryzias latipes)
Source: BMC Genomics. 2023 Aug 21;24:472. doi: 10.1186/s12864-023-09557-1 (PMC10441761; doi:10.1186/s12864-023-09557-1)
Supplement: Supplementary file 6 — Additional file 6: Figure S6. Distribution pattern of histone modifications around ATAC-seq peaks. [file 12864_2023_9557_MOESM6_ESM.pdf]

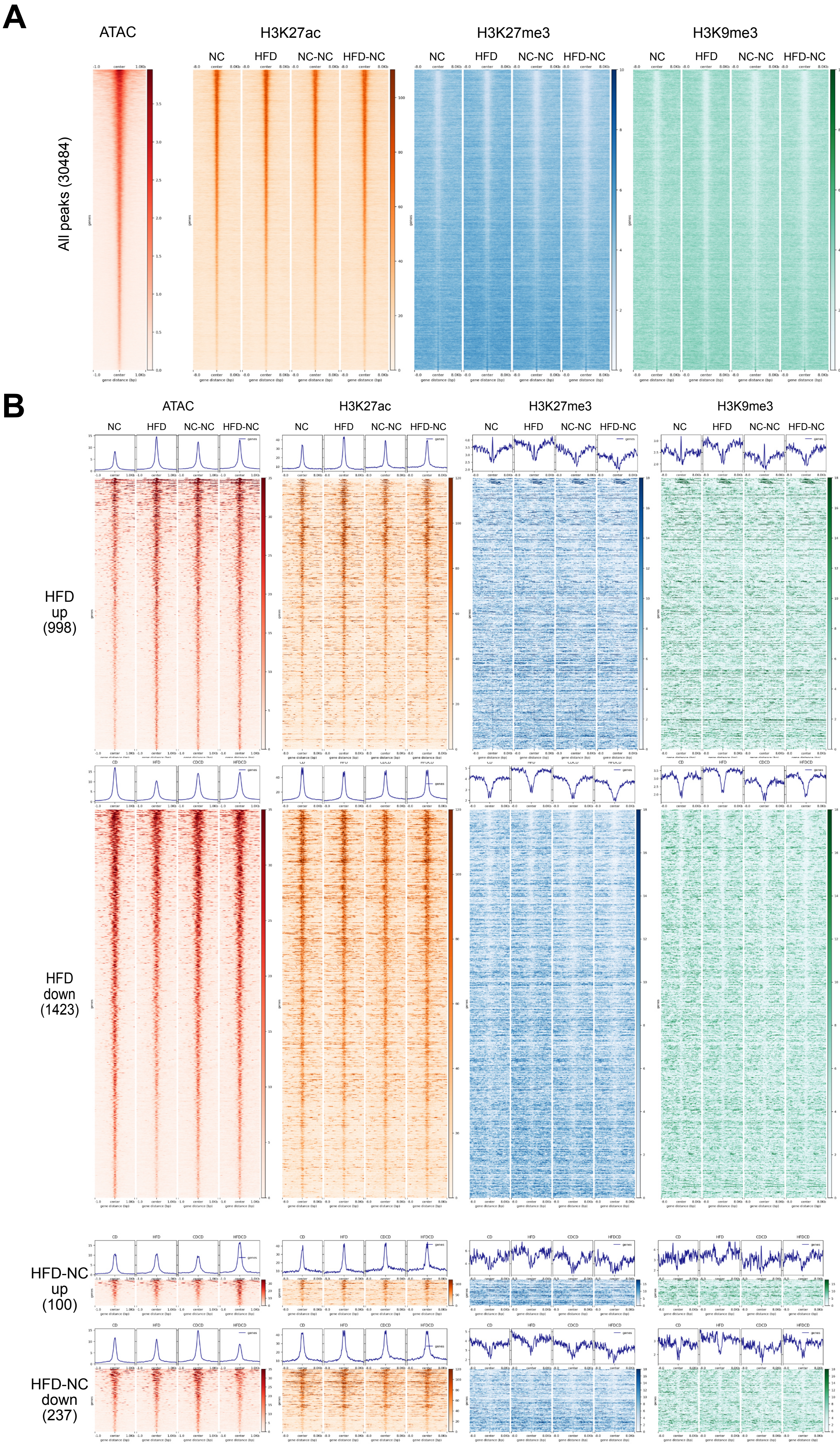
A
ATAC
H3K27ac
H3K27me3
H3K9me3
NC
HFD
NC-NC
HFD-NC
All peaks (30484)
B
HFD up (998)
HFD down (1423)
HFD-NC up (100)
HFD-NC down (237)

Figure S6: Distribution pattern of histone modifications around ATAC-seq peaks.

(A) Distribution pattern of H3K27ac (orange), H3K27me3 (blue), and H3K9me3 (green) around all 30,484 ATAC-seq peaks, sorted by ATAC-seq signal intensity. For ATAC-seq, signal intensities within a 1 kb window from the centers of ATAC-seq peaks are displayed. For histone modifications, signal intensities within an 8 kb window from the centers of ATAC-seq peaks are displayed. Note the positive correlation of ATAC-seq signal with H3K27ac, and the negative correlation with H3K27me3 and H3K9me3 signal. **(B)** Distribution patterns of each histone modification around differentially accessible peaks. For peaks differentially accessible between HFD and NC fish, a moderate positive correlation was observed for H3K27ac. However, the correlation was not observed for differentially accessible peaks between HFD-NC and NC-NC fish. For H3K27me3 and H3K9me3, little changes were seen for differentially accessible peaks at any time point.
