# Supplementary material for: High-fat diet in early life triggers both reversible and persistent epigenetic changes in the medaka fish (Oryzias latipes)
Source: BMC Genomics. 2023 Aug 21;24:472. doi: 10.1186/s12864-023-09557-1 (PMC10441761; doi:10.1186/s12864-023-09557-1)
Supplement: Supplementary file 7 — Additional file 7: Figure S7. Differentially enriched H3K27ac peaks by HFD feeding. [file 12864_2023_9557_MOESM7_ESM.pdf]

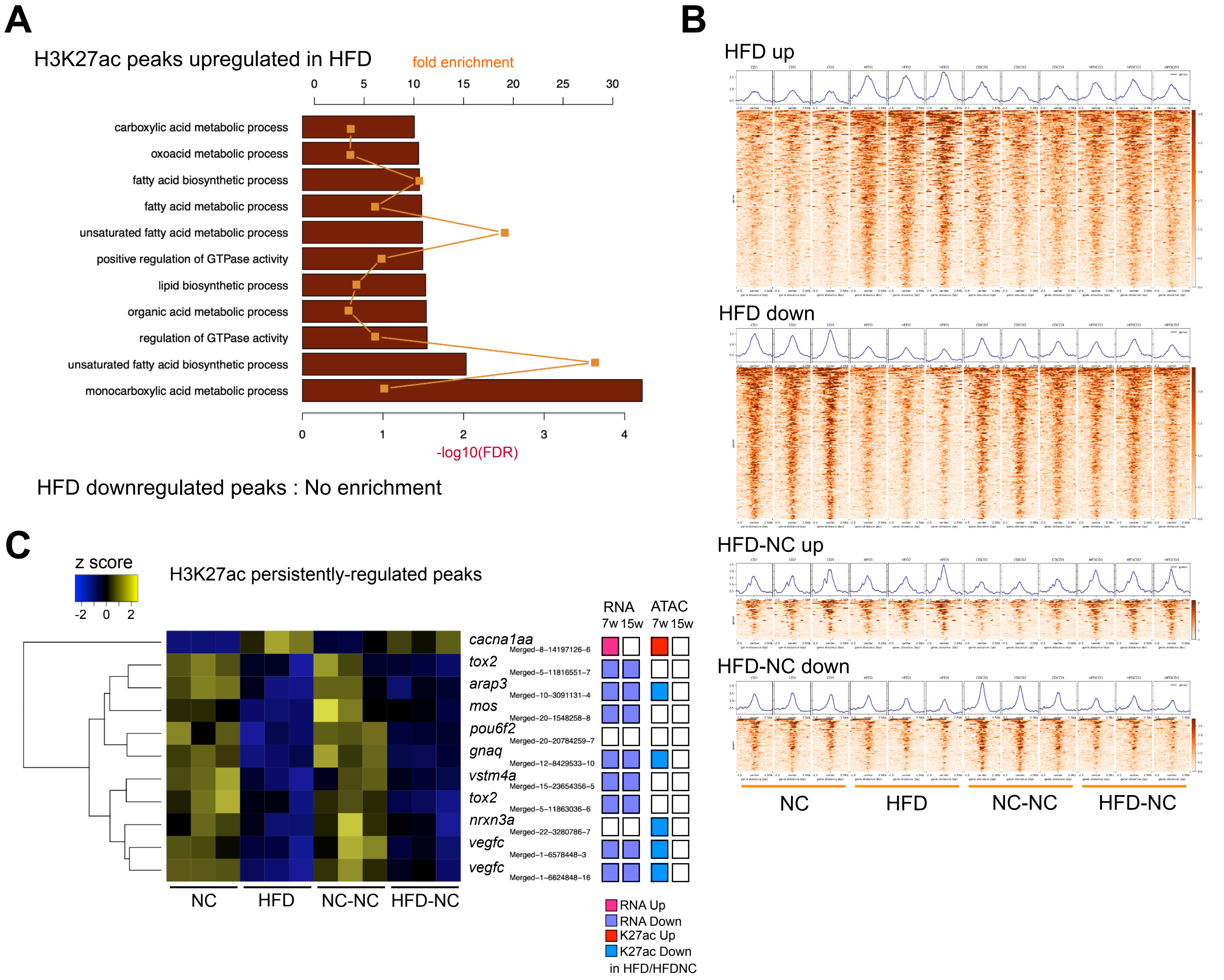

Figure S7: Differentially enriched H3K27ac peaks by HFD feeding.

(A) Gene ontology analyses of genes close to H3K27ac peaks with different enrichment between HFD and NC group fish, inferred by PANTHER v17.0. **(B)** Heatmaps of H3K27ac peaks differentially enriched at seven and 15 weeks of age. Signal intensities in ±2.5 kb regions from the centers of H3K27ac peaks are displayed. **(C)** A heatmap of persistent H3K27ac ChIP-seq peaks after NC (11 peaks in total). Log2-transformed, and Z-transformed, DESeq2 normalized read counts at each peak were displayed. DESeq2 results of RNA-seq and ATAC-seq of nearby genes/peaks were displayed on the right.
