# Supplementary material for: High-fat diet in early life triggers both reversible and persistent epigenetic changes in the medaka fish (Oryzias latipes)
Source: BMC Genomics. 2023 Aug 21;24:472. doi: 10.1186/s12864-023-09557-1 (PMC10441761; doi:10.1186/s12864-023-09557-1)
Supplement: Supplementary file 9 — Additional file 9: Figure S8. H3K9me3 domains downregulated by HFD feeding. [file 12864_2023_9557_MOESM9_ESM.pdf]

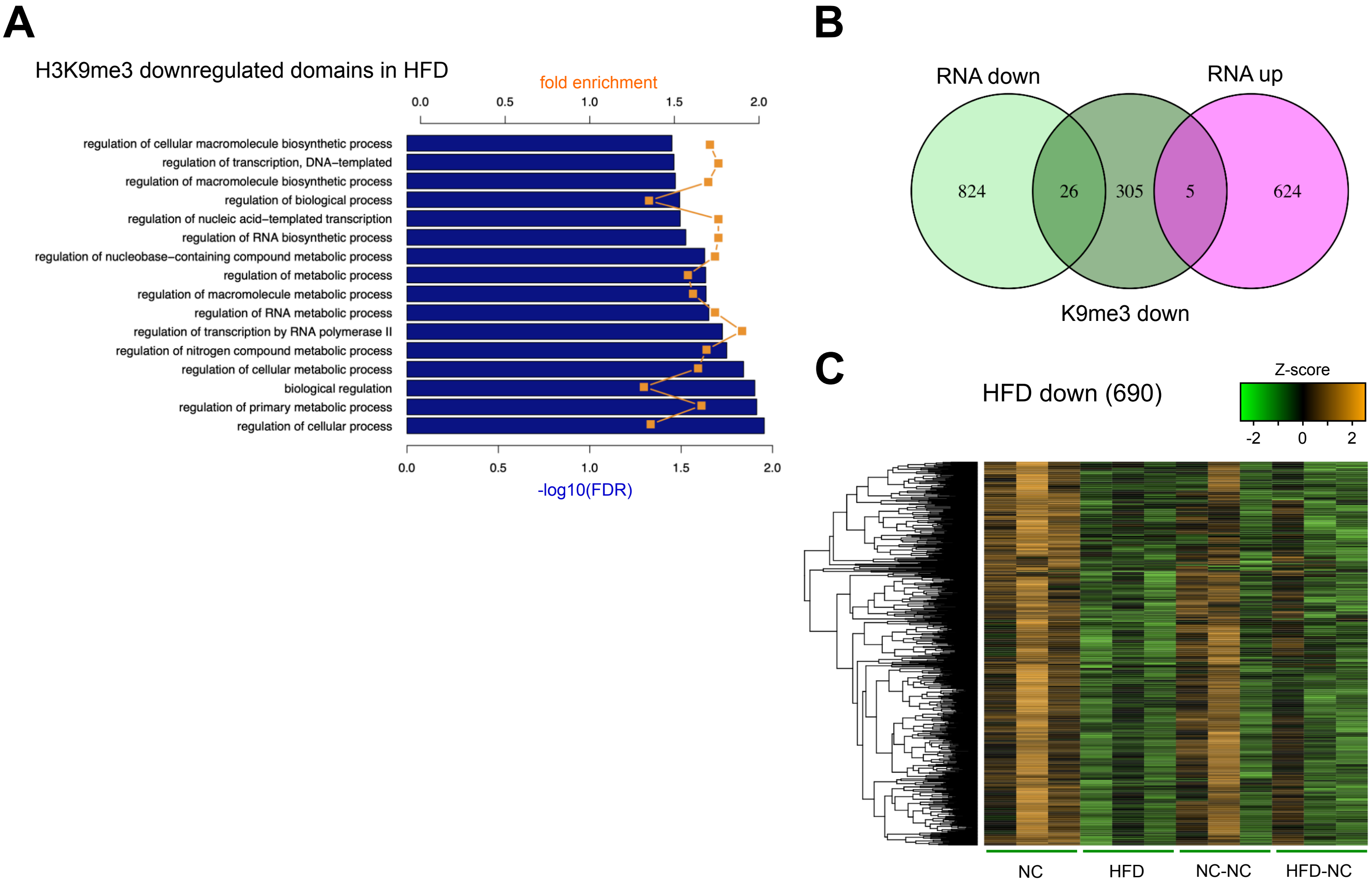

Figure S8: H3K9me3 domains downregulated by HFD feeding.

(A) Gene ontology analyses of genes close to H3K9me3 bins differentially enriched between HFD and NC group fish. **(B)** Venn diagram of genes close to H3K9me3-downregulated bins and genes differentially expressed after HFD feeding. **(C)** A heatmap of H3K9me3 levels for the downregulated bins by HFD. Log2-transformed, and Z-transformed, DESeq2 normalized read counts of H3K9me3 ChIP-seq at each bin are displayed.
