# Supplementary material for: High-fat diet in early life triggers both reversible and persistent epigenetic changes in the medaka fish (Oryzias latipes)
Source: BMC Genomics. 2023 Aug 21;24:472. doi: 10.1186/s12864-023-09557-1 (PMC10441761; doi:10.1186/s12864-023-09557-1)
Supplement: Supplementary file 10 — Additional file 10: Figure S9. Motif analysis of ATAC-seq peaks with decreased accessibility at 15 weeks of age. [file 12864_2023_9557_MOESM10_ESM.pdf]

ATAC-seq HFD-NC down (237 peaks, p-value < 0.01, DESeq2)

Rank	Motif	P-value	log P-pvalue	% of Targets	% of Background	STD(Bg STD)	Best Match/Details
1	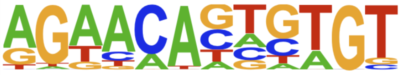	1e-12	-2.881e+01	23.63%	8.12%	143.5bp (202.9bp)	GRE(NR),IR3/RAW264.7-GRE-ChIP-Seq(Unpublished)/Homer(0.876) More Information | Similar Motifs Found

Figure S9: Motif analysis of ATAC-seq peaks with decreased accessibility at 15 weeks of age.

De novo motif analysis of 237 ATAC-seq peaks with decreased accessibility at 15 weeks of age, inferred by HOMER v4.11.
