# Supplementary material for: High-fat diet in early life triggers both reversible and persistent epigenetic changes in the medaka fish (Oryzias latipes)
Source: BMC Genomics. 2023 Aug 21;24:472. doi: 10.1186/s12864-023-09557-1 (PMC10441761; doi:10.1186/s12864-023-09557-1)
Supplement: Supplementary file 11 — Additional file 11: Figure S10. Track views of persistent peaks. [file 12864_2023_9557_MOESM11_ESM.pdf]

A

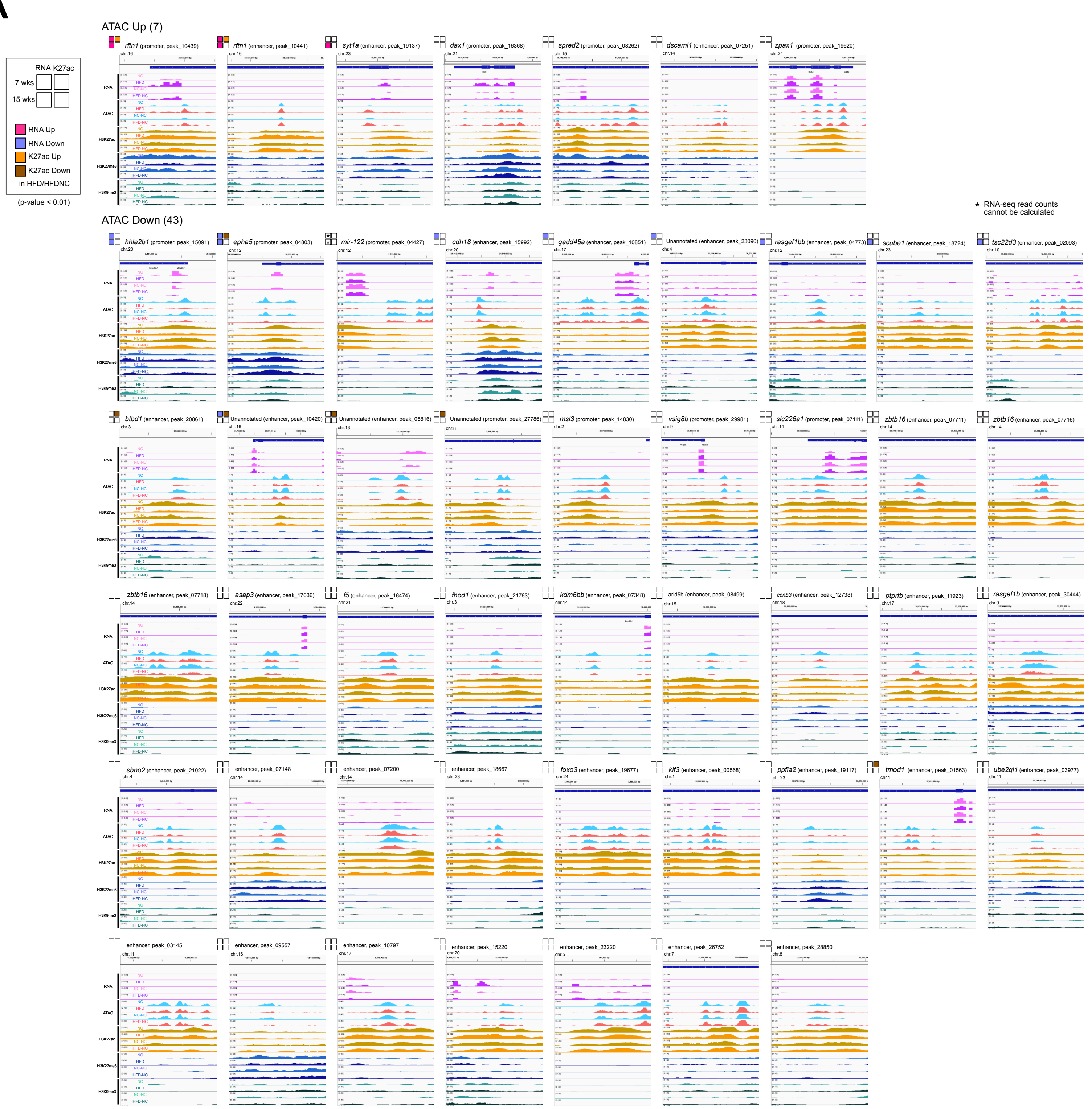
ATAC Up (7)
RNA K27ac
7 wks
15 wks
RNA Up
RNA Down
K27ac Up
K27ac Down
in HFD/HFDNC
(p-value < 0.01)
rftn1 (promoter, peak_10439)
rftn1 (enhancer, peak_10441)
syt1a (enhancer, peak_19137)
dax1 (promoter, peak_16368)
spred2 (promoter, peak_08262)
dscaml1 (enhancer, peak_07251)
zpax1 (promoter, peak_19620)
RNA
ATAC
H3K27ac
H3K27me3
H3K9me3
NC
HFD
NC-NC
HFD-NC
* RNA-seq read counts cannot be calculated
ATAC Down (43)
hhla2b1 (promoter, peak_15091)
epha5 (promoter, peak_04803)
mir-122 (promoter, peak_04427)
cdh18 (enhancer, peak_15992)
gadd45a (enhancer, peak_10851)
Unannotated (enhancer, peak_23090)
rasgef1bb (enhancer, peak_04773)
scube1 (enhancer, peak_18724)
tsc22d3 (enhancer, peak_02093)
btbd1 (enhancer, peak_20861)
Unannotated (enhancer, peak_10420)
Unannotated (enhancer, peak_05816)
Unannotated (promoter, peak_27786)
msl3 (promoter, peak_14830)
vsig8b (promoter, peak_29981)
slc226a1 (promoter, peak_07111)
zbtb16 (enhancer, peak_07711)
zbtb16 (enhancer, peak_07716)
zbtb16 (enhancer, peak_07718)
asap3 (enhancer, peak_17636)
f5 (enhancer, peak_16474)
fhod1 (enhancer, peak_21763)
kdm6bb (enhancer, peak_07348)
arid5b (enhancer, peak_08499)
ccnb3 (enhancer, peak_12738)
ptprfb (enhancer, peak_11923)
rasgef1b (enhancer, peak_30444)
sbno2 (enhancer, peak_21922)
enhancer, peak_07148
enhancer, peak_07200
enhancer, peak_18667
foxo3 (enhancer, peak_19677)
klf3 (enhancer, peak_00568)
ppfia2 (enhancer, peak_19117)
tmod1 (enhancer, peak_01563)
ube2ql1 (enhancer, peak_03977)
enhancer, peak_03145
enhancer, peak_09557
enhancer, peak_10797
enhancer, peak_15220
enhancer, peak_23220
enhancer, peak_26752
enhancer, peak_28850

B

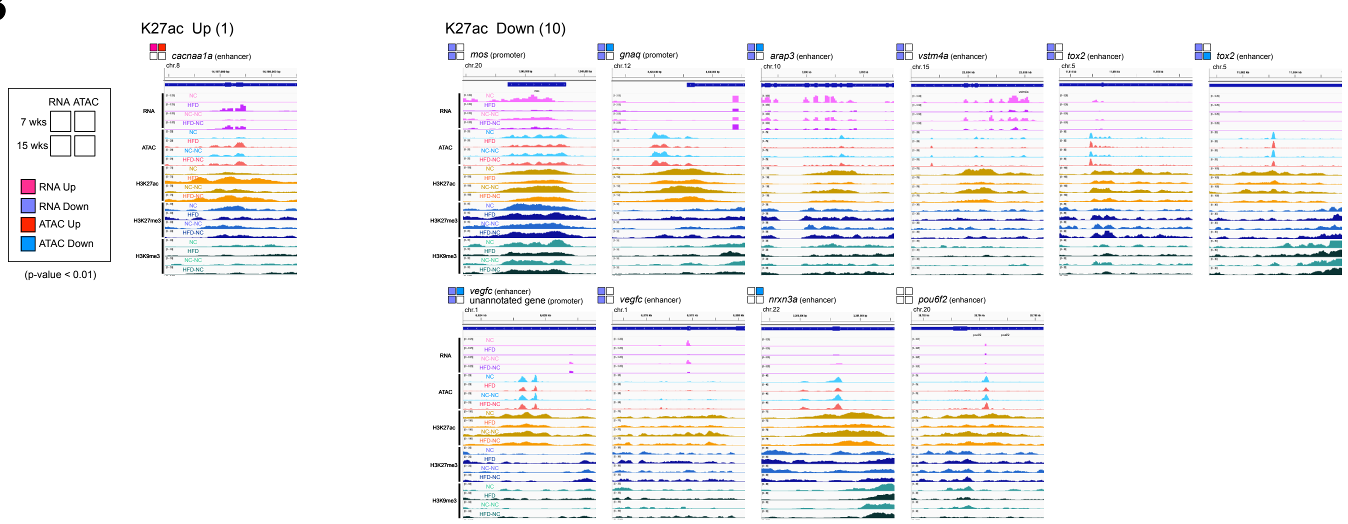
K27ac Up (1)
K27ac Down (10)
RNA ATAC
7 wks
15 wks
RNA Up
RNA Down
ATAC Up
ATAC Down
(p-value < 0.01)
cacnaa1a (enhancer)
mos (promoter)
gnaq (promoter)
arap3 (enhancer)
vstm4a (enhancer)
tox2 (enhancer)
tox2 (enhancer)
vegfc (enhancer)
unannotated gene (promoter)
vegfc (enhancer)
nrxn3a (enhancer)
pou6f2 (enhancer)
RNA
ATAC
H3K27ac
H3K27me3
H3K9me3
NC
HFD
NC-NC
HFD-NC

Figure S10: Track views of persistent peaks.

(A) Track views of the 50 persistent ATAC-seq peaks. DESeq2 results of RNA-seq and H3K27ac ChIP-seq of nearby genes/peaks are displayed on the upper left. **(B)** Track views of the 11 persistent H3K27ac ChIP-seq peaks. DESeq2 results of RNA-seq and ATAC-seq of nearby genes/peaks are displayed on the upper left.
