# Supplementary material for: High-fat diet in early life triggers both reversible and persistent epigenetic changes in the medaka fish (Oryzias latipes)
Source: BMC Genomics. 2023 Aug 21;24:472. doi: 10.1186/s12864-023-09557-1 (PMC10441761; doi:10.1186/s12864-023-09557-1)
Supplement: Supplementary file 12 — Additional file 12: Figure S11. Track views of ATAC-seq peaks changing to opposite direction after a switch to NC. [file 12864_2023_9557_MOESM12_ESM.pdf]

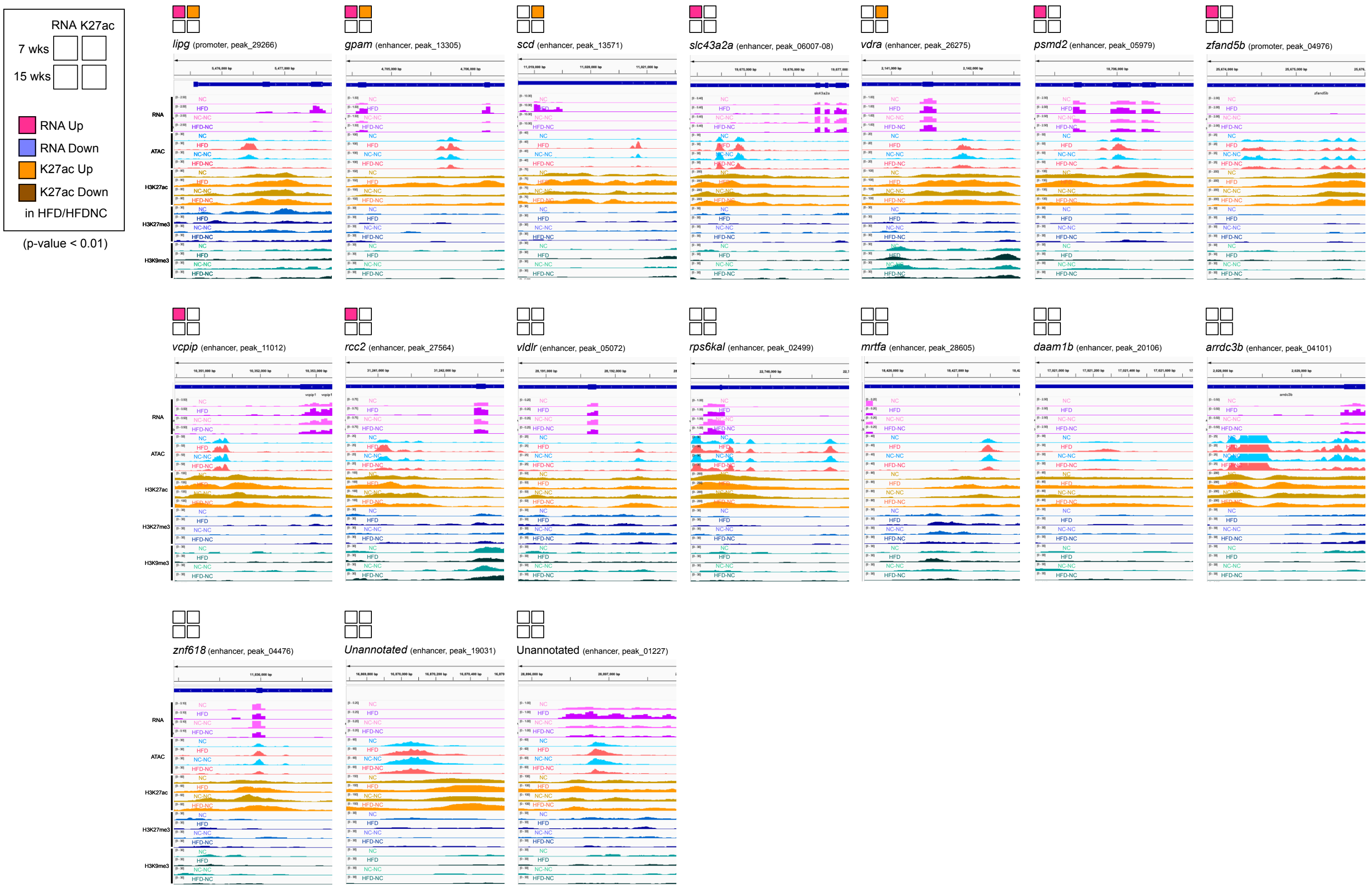

Figure S11: Track views of ATAC-seq peaks changing to opposite direction after a switch to NC.

Track views of the 18 ATAC-seq peaks showing increased chromatin accessibility by HFD but decreased accessibility after a switch to NC. DESeq2 results of RNA-seq and H3K27ac ChIP-seq of nearby genes/peaks are displayed on the upper left.
