# Supplementary material for: High-fat diet in early life triggers both reversible and persistent epigenetic changes in the medaka fish (Oryzias latipes)
Source: BMC Genomics. 2023 Aug 21;24:472. doi: 10.1186/s12864-023-09557-1 (PMC10441761; doi:10.1186/s12864-023-09557-1)
Supplement: Supplementary file 15 — Additional file 15: Figure S12. Rescaling procedure of read counts for visualization of ChIP-seq and ATAC-seq. [file 12864_2023_9557_MOESM15_ESM.pdf]

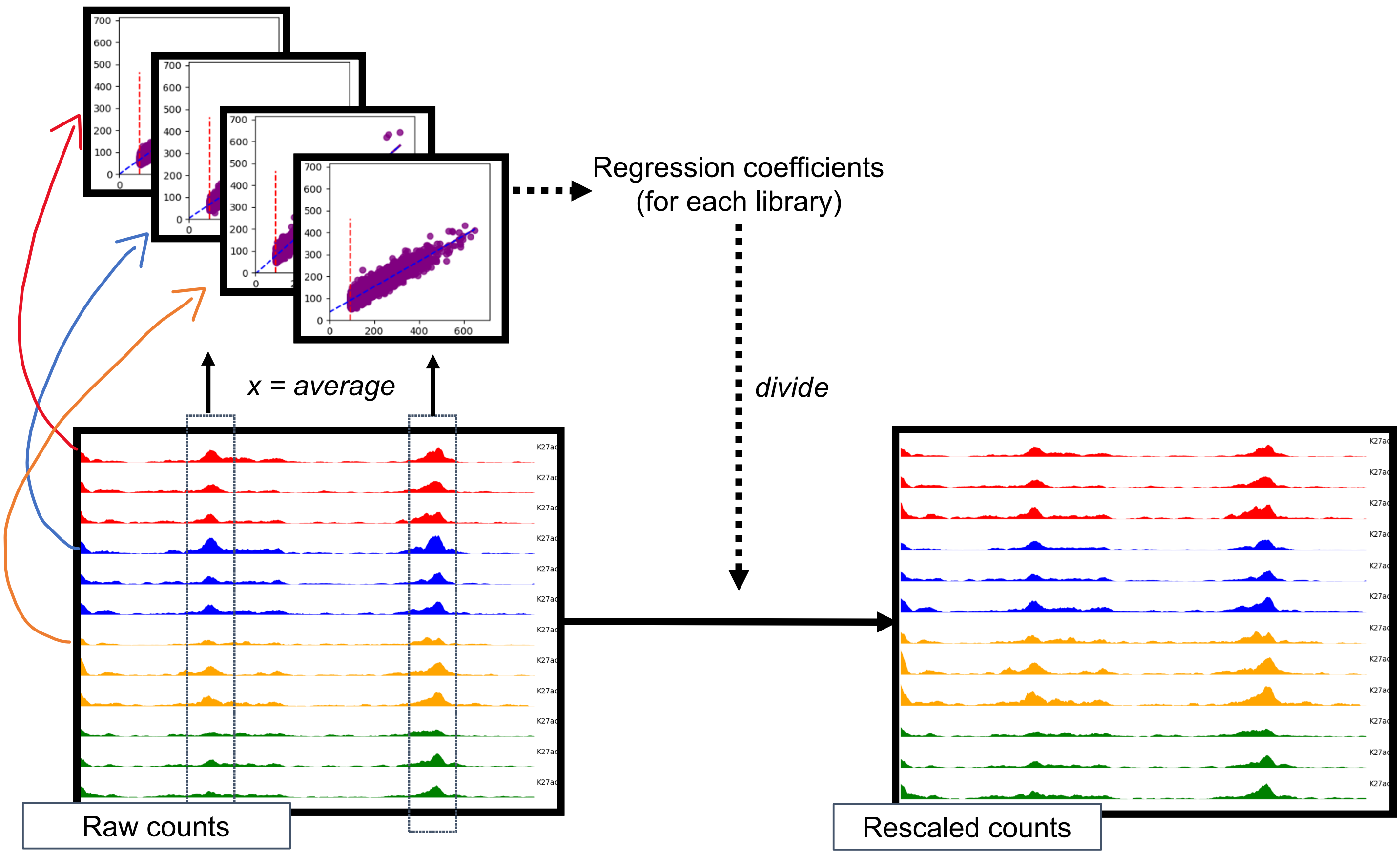

Figure S12: Rescaling procedure of read counts for visualization of ChIP-seq and ATAC-seq. The top left panels describe the regression process. Each point in these panels represents read count data from a single region. For each library, the signals on the selected regions (y-axis) were regressed on the averages of all libraries (x-axis). In this process, the regions whose average signal was within the top 100 or below the global average (average across all regions and libraries) were excluded (red dotted lines). Then, the raw counts (left bottom panel) were divided by the corresponding regression coefficients (blue dotted lines) to obtain the rescaled counts (the right bottom panel).
